# The roles and diagnostic value of miRNA-1246 in the serum of patients with intracranial aneurysms

**DOI:** 10.1515/tnsci-2022-0227

**Published:** 2022-07-07

**Authors:** Haijie Jiang, Yansheng Ding, Lili Wu, Chunyan Jiang, Chengdong Wang

**Affiliations:** Department of Medical Laboratory, Weifang Medical University, Molecular Biological Diagnosis of Cerebrovascular Disease, Weifang, China; Clinical Laboratory, Weifang People’s Hospital, Diagnosis by Clinical Examination, Weifang, China; Clinical laboratory, Weifang People’s Hospital, Weifang, China; Neurology department, Weifang People’s Hospital, Weifang, China

**Keywords:** intracranial aneurysm, microRNAs, diagnosis, bioinformatics

## Abstract

**Background:**

Inflammatory response is one of the important factors affecting the formation of intracranial aneurysm. miR-1246 is involved in the regulation of several inflammatory diseases; however, its expression levels and the mechanism of action in intracranial aneurysms remain further investigated.

**Methods:**

Bioinformatics was used to analyze the levels of micro-RNAs (miRNAs) in the serum of intracranial aneurysm patients as well as in the intracranial aneurysm tissues downloaded from the GEO RNA-seq database. Blood samples were collected pre-operatively from patients with intracranial aneurysms as well as from healthy volunteers, and miRNA-1246 expression levels were detected using quantitative reverse transcriptase polymerase chain reaction. Meanwhile, the diagnostic value of miR-1246 for intracranial aneurysm was explored using the receiver operating characteristic (ROC) curve.

**Principle findings and results:**

Serum levels of miR-1246 were elevated in intracranial aneurysm patients. Bioinformatics studies revealed that the target genes of miR-1246, TP53, glycogen synthetase kinase (GSK), and transcription factor YY1 may play important roles in the development of intracranial aneurysms. miR-1246 is involved in inflammatory response, lipid, and atherosclerotic signaling pathways.

**Conclusions and significance:**

High level of miR-1246 is found in the serum of patients with intracranial aneurysms and may serve as a diagnostic or/and treatment marker for intracranial aneurysms.

## 1 Introduction

Intracranial aneurysm is one of the most dangerous vascular diseases of the brain. Its sudden rupture of intracranial aneurysms is the main cause of non-traumatic subarachnoid hemorrhage, which can result in severe damage to the central nervous system as well as to several other organs. Because most intracranial aneurysms are asymptomatic before rupture, early detection of intracranial aneurysms can be effective for monitoring their progression and taking preventive measures. Digital subtraction angiography (DSA) is currently the best approach for diagnosing intracranial aneurysms, but DSA is not suitable for early screening and treatment of intracranial aneurysms since it is invasive and does not show the distal vessels of the occlusion, and in addition, it is costly and potentially risky to visualize the location of the vascular lesion by injecting iodine contrast. Recently, the detection of micro-RNA (miRNA) in circulating immune cells has been used as a means to screen for diagnostic markers of vascular diseases such as intracranial aneurysms, abdominal aortic aneurysms, and stroke. miRNAs are known to inhibit protein translation by regulating the 3′ untranslated region of target gene mRNAs and are involved in important biological processes such as apoptosis and metabolism in the organism [1]. Several miRNAs have been previously identified to be associated with the development and progression of intracranial aneurysms and can have an impact on the processes of angiogenesis and extracellular matrix degradation and on the biological processes such as inflammatory responses and apoptosis of vascular smooth muscle cells (VSMC) through various pathways. The formation and development of intracranial aneurysms are the results of endothelial dysfunction and conversion of the VSMC phenotype to a pro-inflammatory phenotype. miR-409-3p is a widely studied miRNA in inflammatory diseases, and it triggers the inflammatory response mainly by targeting Nr4a2 to activate the NF-κB pathway [2].

In this study, we used second-generation gene sequencing to screen for differentially expressed miRNAs associated with inflammation in the serum of patients with intracranial aneurysms. qRT-PCR was used to detect the serum levels of miRNAs in patients with intracranial aneurysms and healthy individuals. Bioinformatics was used to analyze the differentially expressed miRNAs and to explore the significance of inflammatory response in intracranial aneurysms and the diagnostic value of inflammation-related miRNAs in intracranial aneurysms.

## 2 Materials and methods

### 2.1 Study subjects and sample collection

Patients who attended the Neurosurgery Department at the Weifang People’s Hospital in the Shandong Province from September 2020 to December 2021 were diagnosed with intracranial aneurysm and selected for the study, while healthy individuals in the Health Management Center were selected as healthy controls. The inclusion criteria for the group of healthy individuals were the following: (i) matched to patients in terms of age, gender, and other aspects of medical history; and (ii) healthy individuals who did not have inflammation-related diseases, cerebrovascular-related diseases, history of various types of tumors, and immune diseases. The inclusion criteria for the group of patients with intracranial aneurysms were the following: (i) patients presenting with sudden onset of headache, ptosis, dizziness or diplopia, and diagnosed by DSA; and (ii) patients with various inflammatory-related diseases, cerebrovascular-related diseases, history of various tumors, and immune diseases other than intracranial aneurysms were excluded.

Three (3) mL of venous blood was collected from the patient’s pre-operative elbow vein (fasting for more than 8 h), and blood from healthy controls was also collected at the same time. The blood samples were immediately centrifuged at 3,500 rpm for 5 min after collection. The serum was collected in two 1.5 mL RNAase-free centrifuge tubes, sealed and numbered, and immediately stored in a −80°C freezer.


**Ethical approval:** The research related to human use has been complied with all the relevant national regulations, institutional policies and in accordance with the tenets of the Helsinki Declaration, and has been approved by the Medical Ethics Committee of the Weifang People’s Hospital.
**Informed consent:** Informed consent has been obtained from all individuals included in this study.

### 2.2 Screening and bioinformatics analysis of target miRNAs

There were two main data sources in this study including (i) the second-generation gene sequencing (RNA-seq) data for the blood levels of miRNAs in patients with intracranial aneurysms; (ii) the data of differentially expressed miRNAs in tissues of patients with intracranial aneurysms were obtained and screened from GEO database. Second-generation gene sequencing to determine the levels of miRNA in the serum of the patients with intracranial aneurysms was partially conducted by researchers from BGI Genomics Co., Ltd. The GSE46336 and GSE66239 data sets were downloaded from the GEO database of the National Center for Biotechnology Information (NCBI) and contained 10 intracranial aneurysm samples and 13 control samples (middle cerebral artery tissue and superficial temporal artery tissue). GSE46336 and GSE66239 expression profiles were analyzed using GEO2R tool, and the screening criteria were *P* < 0.05, the absolute value of log_2_FC > 1.5. After the screening, differentially expressed microRNAs (DEMs) were identified from the overlap using Venn diagrams, and those with differential expression in both tissues and serum and consistent expression were considered as differentially expressed miRNAs. The miRNet database (https://www.mirnet.ca/), a comprehensive miRNAs target gene database, was used to predict the target genes of the screened differentially expressed miRNAs in the aneurysm tissues of intracranial aneurysm patients, and the target genes of the screened highly expressed specific miRNAs were transcribed factor annotation by FunRich (v3.3.1) software. Transcription factors that met *P* < 0. 05 were used as entry factors for further analysis. gene ontology (GO) enrichment analysis and kyoto encyclopedia of genes and genomes (KEGG) signaling pathway prediction were performed using the DAVID database (https://david.ncifcrf.gov/) for the screened target genes with high expression specificity for DEMs. The criterion for screening was *P* < 0.05. The obtained DEMs of target genes were uploaded to a string database (https://string-db.org/) to predict the relationship between the target genes and then visualized using Cytoscape software (https://cytoscape.org/) to find the top five genes by various computational methods including the top five. The core target genes located in the top 50 were also calculated using the degree of connectivity search. CytoHubba sorts the nodes by several topological algorithms based on the characteristics of the pre-loaded protein protein interaction (PPI) network.

### 2.3 Determine miRNA levels in serum

The miRNA was extracted using the Easy Pure miRNA kit. 200 μL of serum was mixed with 1 mL lysis buffer for 30 s and then incubated at room temperature for 5 min before adding nematode outer reference solution, and the solution was mixed thoroughly and left for 10 min. Chloroform (200 μL) was then added to the serum and mixed well, and the mixture was centrifuged at 10,000×*g* for 15 min at 4°C; the upper aqueous phase was aspirated to another RNAase-free centrifuge tube. The volume of the aqueous phase was measured accurately, and one-third volume of anhydrous ethanol was added to the aqueous phase and mixed well. The obtained solution was transferred to the RNA Spin Column which was centrifuged at 12,000×*g* for 30 s at room temperature and the effluent was retained. The effluent was transferred to a clean 2-mL RNAase-free centrifuge tube, and 1.25 times the volume of effluent was mixed with anhydrous ethanol. The obtained solution was transferred to a miRNA Spin Column and centrifuged at 12,000×*g* for 30 s at room temperature, and then, the effluent was discarded (the volume of solution is larger than the volume of the miRNA Spin Column; this step is repeated until the entire solution is added). Wash buffer (500 μL) was added to the miRNA Spin Column, centrifuged at room temperature at 12,000×*g* for 30 s, and then, the effluent was discarded. The procedure was repeated once. miRNA Spin Column was centrifuged at room temperature at 12,000×*g* for 2 min in order to completely remove the residual ethanol from it. The miRNA Spin Column was placed into another 1.5 mL RNAase-free centrifuge tube, and 35 μL of RNAase-free water was added from the center of the miRNA Spin Colum, and then, it was allowed to stand at room temperature for 1 min. The miRNA Spin Column was centrifuged for 1 min at 12,000×*g* at room temperature to elute the miRNA. The tubes were sealed and numbered, and the extracted miRNA was directly reverse-transcribed to cDNA or stored in an −80°C freezer. The miRNA was used as a template for reverse transcription to synthesize cDNA. The reactions include the following: 10 μL of 2× Transcript miRNA reaction mix, 9 μL of extracted miRNA template, and 1 μL of Transcript miRNA RT enzyme mix. The reverse transcription reaction was performed at 37°C for 1 h and then heated to 85°C for 5 s temperature. The quantitative reverse transcriptase-polymerase chain reaction (qRT-PCR) includes the following: 10 μL of 2× Transtart Tip Green qPCR Super mix, 6 μL of five-fold diluted cDNA template, 2 μL of forward primer, and 2 μL of reverse primer. The qPCR was performed under the following conditions: 95°C for 30 s (1 cycle), 95°C for 5 s, and 60°C for 30 s (45 cycles). For spiking, two replicate wells were added for each cDNA. After waiting for the amplification reaction to be completed, the relative quantification method (with cel-miR-39 as a reference) was used to determine the miRNA level in the patient’s serum. The formula was used for calculation as 2^−ΔCt^ (ΔCt = Ct_target miRNA_ − Ct_cel-miR-39_). The Ct values of each target miRNA and cel-miR-39-3p for each patient were used as the average of the Ct values of the two replicate wells. The reagents used in this experiment were obtained from the Beijing TransGen Biotech Co., Ltd.

### 2.4 Statistical methods

SPSS 26.0 software as well as GraphPad Prism 9 was used to analyze the data. The normality of the data was tested using the Shapiro–Wilk test. Normally distributed data were expressed as mean ± standard deviation (Mean ± SD), and non-normally distributed data were expressed as median and quartiles M (P25 and P75). The non-parametric Mann–Whitney *U* test was used to analyze whether there were differences in the data distribution of serum miR-1246 levels in the intracranial aneurysm group and healthy control group. ROC curves were used to assess the diagnostic value of miRNA in the serum of patients with intracranial aneurysms. The difference was considered statistically significant by statistical analysis *P* < 0.05.

## 3 Results

### 3.1 Clinical profile of patients with intracranial aneurysm

A total of 58 patients with intracranial aneurysms were included in this study, including 7 cases of multiple intracranial aneurysms and 51 cases of single intracranial aneurysms. There were 23 male patients and 35 female patients in the intracranial aneurysm group. A total of 44 healthy individuals were included in the study. There were 23 male patients and 21 female patients in the healthy group. The difference in the composition ratio between male and female patients was not statistically significant between the intracranial aneurysm group and the healthy control group (*P* = 0.130). The average age of the intracranial aneurysm group was 55.02 ± 9.03 years, and the age of the healthy control group was 55.30 ± 10.03 years. The difference in age between the intracranial aneurysm group and the healthy control group was not statistically significant (*P* = 0.574 (Table 1)).

**Table 1:** Clinical information of the intracranial aneurysm patient group and healthy control group

	Intracranial aneurysm group (*n* = 58)	Health group (*n* = 44)	*P-*Value
Gender	Male (*n* = 23)	Male (*n* = 23)	0.205^b^
	Female (*n* = 35)	Female (*n* = 21)	
Age	55.02 ± 9.03	55.30 ± 10.03	0.883^a^
Multiple intracranial aneurysms	Yes (*n* = 7)		
	No (*n* = 51)		

Note: a – *t*-test; b – chi-square test.

### 3.2 Screening differentially expressed miRNAs

**Figure 1:**
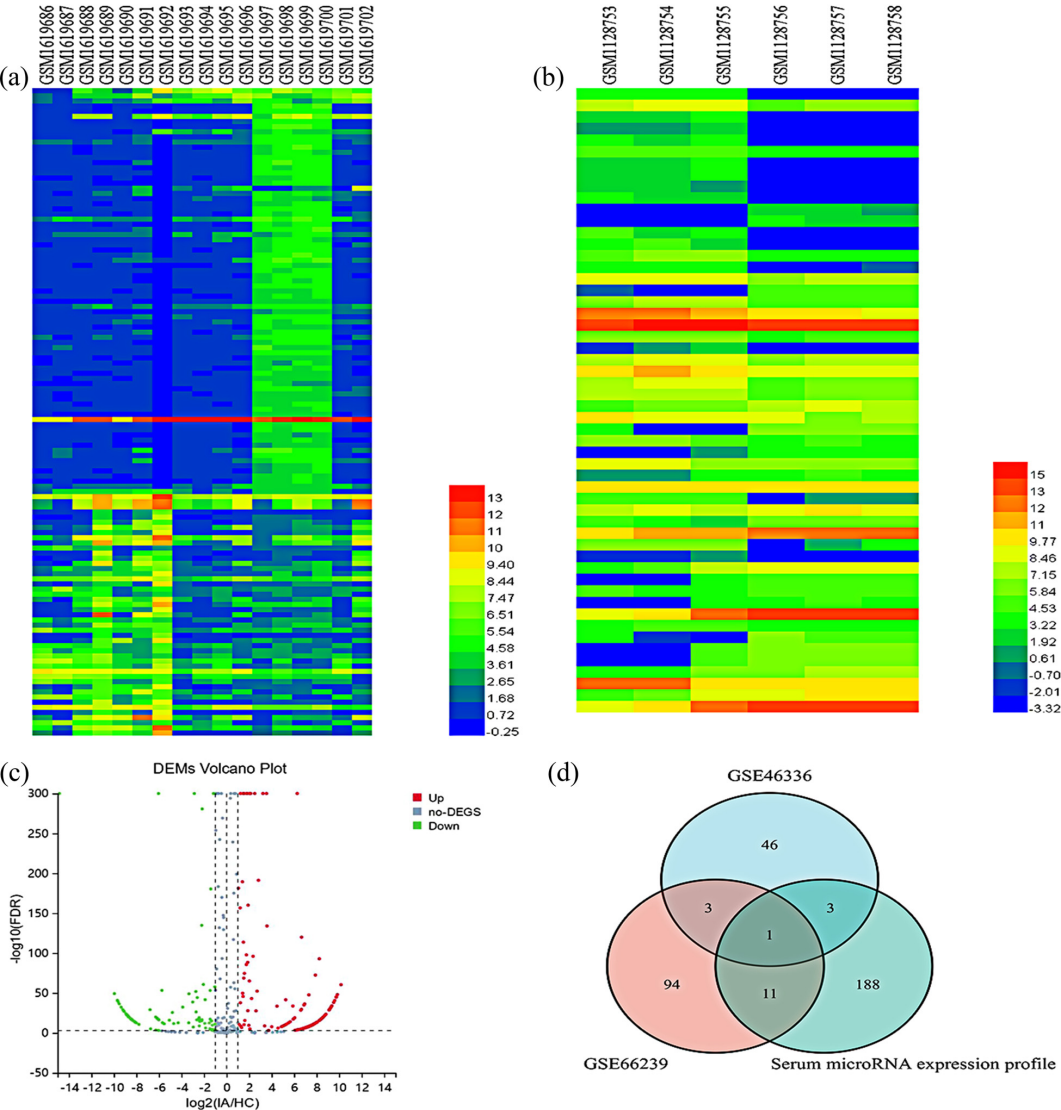
Differentially expressed miRNAs from the screening. (a) Heat map of GSE66239 differentially expressed miRNAs; (b) heat map of GSE46336 differentially expressed miRNAs; (c) volcano plot of differentially expressed miRNAs; and (d) Venn diagram of differentially expressed miRNAs in serum and tissue.

A total of 109 differentially expressed miRNAs were screened and identified from the GSE66239 dataset, among which 35 differentially expressed miRNAs were upregulated and 74 differentially expressed miRNAs were downregulated (Figure 1a); 53 differentially expressed miRNAs were screened and identified from the GSE46336, among which 31 differentially expressed miRNAs were upregulated and 22 differentially expressed miRNAs were downregulated (Figure 1b). The miRNAs whose expression showed a consistent status in both datasets include hsa-miR-1246, hsa-miR-127-3p, hsa-miR-409-3p, and hsa-miR-654-3p, among which hsa-miR-409-3p and hsa-miR-1246 showed a consistent high expression status with log_2_FC values greater than 2 in both datasets. By gene sequencing as well as online software analysis, 203 differentially expressed miRNAs were screened and identified, including 138 miRNAs with upregulated expression levels and 65 miRNAs with downregulated expression levels (Figure 1c). Based on the above results, it was found that miRNA-1246 showed upregulation of expression levels in both tissues and serum (Figure 1d), bioinformatics analysis, and study were performed.

### 3.3 Bioinformatic analysis of miRNA-1246

**Figure 2:**
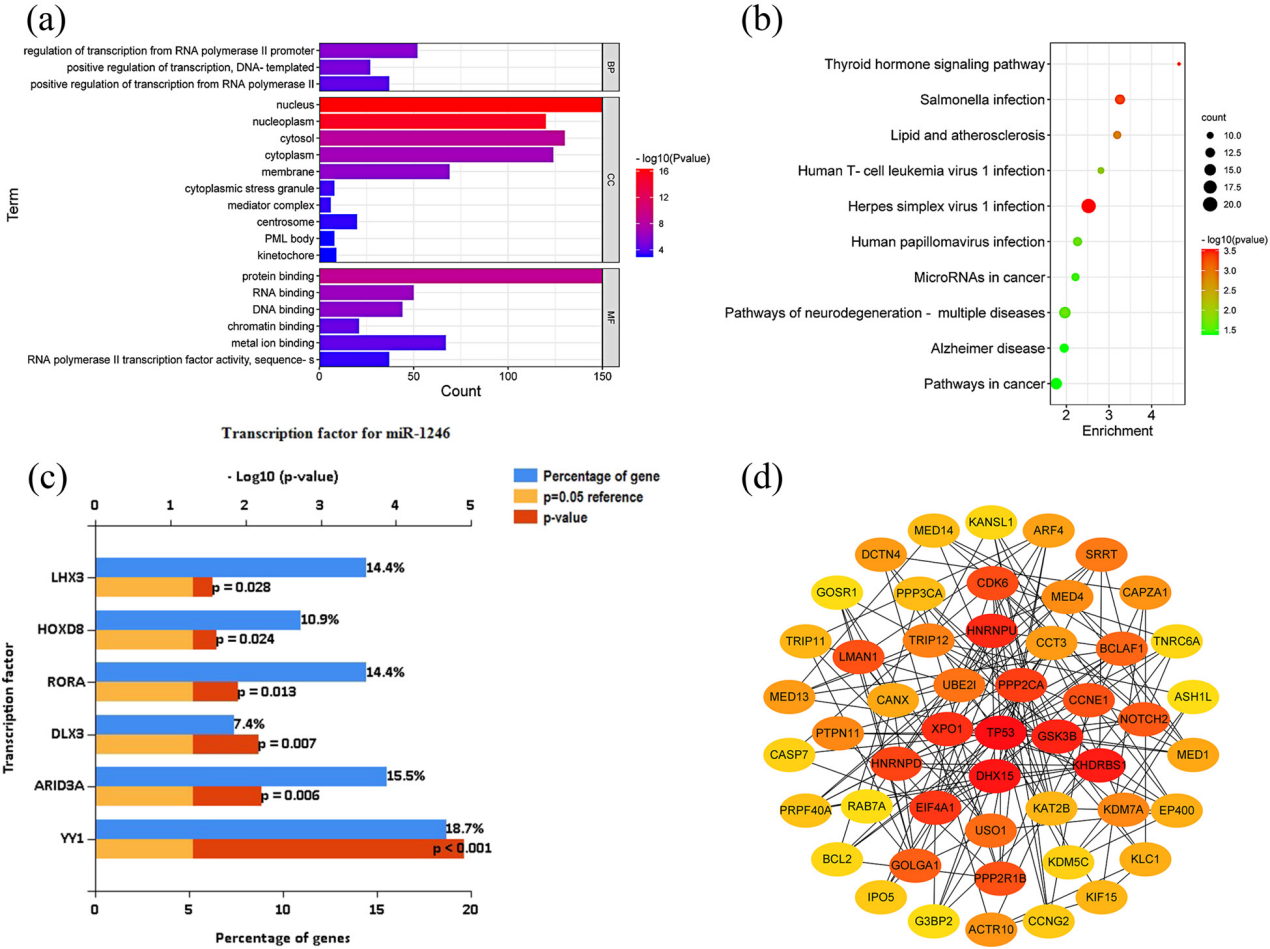
Bioinformatic analysis of miRNA-1246 target genes. (a) GO analysis; (b) KEGG analysis; (c) transcription factor enrichment analysis; and (d) PPI analysis.

A total of 319 target genes regulated by miRNA-1246 were predicted by miRNet database (https://www.mirnet.ca/). By performing transcription factor analysis on the target genes of differentially expressed miRNAs, the six transcription factors with the largest differences were found, including YY1, ARID3A, DLX3, RORA, HOXD8, and LHX3 (Figure 2c). Functional annotation and signaling pathway prediction were performed for 319 target genes of DEMs. The results reveal that the relevant target genes were mainly focused on the pathways such as inflammatory response-related pathways, lipid and atherosclerotic signaling pathways, Alzheimer’s disease, and neurodegenerative disease pathways (Figure 2a and b, Tables 3 and 4). Subsequently, the top five key genes were each taken using different calculation methods of CytoHubba, and the five key genes with the highest number of occurrences in different methods were used as the final key genes, namely, TP53, GSK3B, XPO1, UBE2I, and DHX15 (Table 2 and Figure 2d).

**Table 2:** Top five key genes identified by different calculation methods

Method	MCC	Degree	DMNC	MNC	EPC
Key genes	TP53	TP53	KLC1	TP53	TP53
	DHX15	XPO1	TRIP11	XPO1	XPO1
	KHDRBS1	GSK3B	KIF15	UBE2I	PPP2CA
	GSK3B	UBE2I	MED14	GSK3B	UBE2I
	HNRNPU	DHX15	CAPZA1	PPP2CA	GSK3B

**Table 3:** KEGG target gene enrichment analysis

Pathway	Genes	Fold enrichment
Herpes simplex virus 1 infection	ZNF320, ZNF460, ZNF283, ZNF480, ZNF160, PTPN11, ZNF12, PIK3CB, TAPBP, ZNF717, ZNF627, ZNF85, ZNF548, BCL2, BAX, ZNF557, ZNF841, ZNF763, TP53, ZNF267	2.52
Pathways of neurodegeneration – multiple diseases	GSK3B, XBP1, DCTN4, AXIN2, KLC1, SDHB, PPP3CA, CASP7, PSMC6, BCL2, DVL3, BAX, SLC25A5, CALM2, ACTR10	1.97
Pathways in cancer	NOTCH2, GSK3B, PIK3CB, AXIN2, RASGRP3, CASP7, CDK6, CCNE1, MSH3, PIM1, BCL2, DVL3, BAX, CALM2, TP53	1.76
Salmonella infection	CYFIP1, RAB5B, DCTN4, PIK3CB, KLC1, CASP7, ARPC2, BCL2, BAX, TAB3, ACBD3, ACTR10, RAB7A	3.26
Human papillomavirus infection	NOTCH2, ATP6V1A, PPP2CA, GSK3B, CDK6, PPP2R1B, CCNE1, DVL3, BAX, AXIN2, PIK3CB, TP53	2.26
Alzheimer’s disease	GSK3B, PPP3CA, XBP1, CASP7, PSMC6, DVL3, AXIN2, PIK3CB, SLC25A5, KLC1, CALM2, SDHB	1.95
Lipid and atherosclerosis	GSK3B, PPP3CA, XBP1, CASP7, BCL2, NFATC3, BAX, PIK3CB, SOD2, CALM2, TP53	3.19
MicroRNAs in cancer	NOTCH2, MARCKS, UBE2I, CDK6, CCNE1, RDX, CL2, PIM1, PIK3CB, TP53, TP63	2.21
Human T-cell leukemia virus 1 infection	KAT2B, PPP3CA, XPO1, CCNE1, CANX, NFATC3, BAX, PIK3CB, SLC25A5, TP53	2.81
Thyroid hormone signaling pathway	NOTCH2, KAT2B, GSK3B, MED1, MED14, MED13, PIK3CB, MED4, TP53	4.64

**Table 4:** Gene ontology analysis

Term	Count	*P* value	Group
Regulation of transcription from RNA polymerase II promoter	52	2.41 × 10^−6^	BP
Positive regulation of transcription, DNA-templated	27	1.17 × 10^−5^	BP
Positive regulation of transcription from RNA polymerase II	37	7.31 × 10^−5^	BP
Nucleus	159	4.66 × 10^−17^	CC
Nucleoplasm	120	5.59 × 10^−16^	CC
Cytosol	130	4.58 × 10^−9^	CC
Cytoplasm	124	1.59 × 10^−7^	CC
Membrane	69	1.30 × 10^−6^	CC
Cytoplasmic stress granule	8	3.17 × 10^−4^	CC
Mediator complex	6	4.25 × 10^−4^	CC
Centrosome	20	6.50 × 10^−4^	CC
PML body	8	1.19 × 10^−3^	CC
Kinetochore	9	1.26 × 10^−3^	CC
Protein binding	245	1.50 × 10^−9^	MF
RNA binding	50	3.70 × 10^−7^	MF
DNA binding	44	1.76 × 10^−6^	MF
Chromatin binding	21	4.76 × 10^−5^	MF
Metal ion binding	67	8.06 × 10^−5^	MF
RNA polymerase II transcription factor activity, sequence-s	37	5.56 × 10^−4^	MF

### 3.4 Serum expression levels of miR-1246 in intracranial aneurysm group and healthy control group

**Figure 3:**
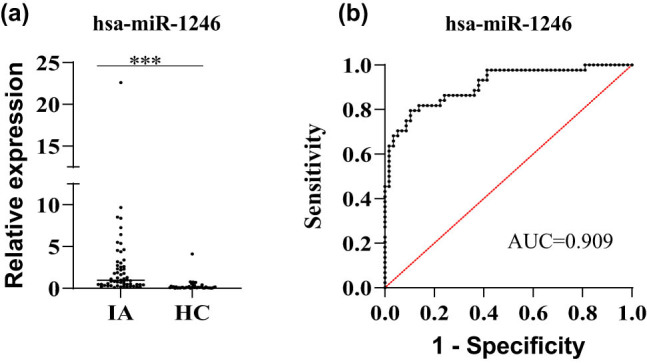
Expression level and ROC diagnostic curve of serum miRNA-1246. (a) Expression level of serum miRNA-1246, (b) ROC diagnostic curve of serum miRNA-1246

The expression levels of miRNA-1246 in serum of healthy control group and aneurysm group were determined using qRT-PCR. The miR-1246 expression level was 0.985 (0.434, 2.815) in the intracranial aneurysm group and 0.116 (0.058, 0.209) in the control group, respectively. Compared with the control group, the miR-1246 level was increased in the serum of intracranial aneurysm group, and the difference was statistically significant (*P* < 0.001) (Figure 3a). The AUC value of serum miR-1246 for the diagnosis of intracranial aneurysm was 0.909 (Figure 3b), and the sensitivity of miR-1246 for the diagnosis of intracranial aneurysm was 79.55%, and the specificity was 89.66%, both with statistically significant differences (*P* < 0.001).

## 4 Discussion

Currently, many studies have found that miR-1246 plays an important role in inflammation-related diseases. miR-1246 provides a new avenue for the diagnosis and treatment of intracranial aneurysms. Alexander described miR-1246 as a “key enhancer” of pro-inflammatory responses in mesenchymal stem cells (MSC) or stromal cells [3]. It was found that miR-1246 is highly expressed in MSCs or stromal cells and that miR-1246 regulates the inflammatory response by directly targeting PRKAR1A and PPP2CB, subunits of PKA and PP2A, and mediates the secretion of CCL2 and CCL5 via activating NF-κB pathway, by which involves the inflammatory response. Bai et al. and colleagues established a rat disease model, stimulated human umbilical vein endothelial cells (HUVEC) using VEGF in order to induce corneal neovascular cells *in vitro*, and performed RNA immunoprecipitation (RIP) [4]. These experiments revealed that miR-1246 in a rat model of corneal neovascularization was suppressed by lncRNA MIAT expression, and it was capable of regulating cell proliferation and migration of HUVEC. It has even been found that miR-1246 is capable of acting on NF-κB signaling pathway and regulating M2 macrophage polarization through NF-κB pathway [5], which confirms Hasan’s study [6]. Hasan and colleagues found that after intracranial aneurysm rupture, there is a change in the ratio of macrophage type M1 to type M2 [6]. NF-κB enhances the high expression of pro-inflammatory molecules such as vascular cell adhesion molecule-1 (VCAM-1), monocyte chemoattractant protien-1 (MCP-1), inducible NO synthase, and matrix metalloproteinases (MMPs) by aneurysmal endothelial cells and macrophages at the transcriptional and translational levels. These inflammatory mediators promote inflammatory cells, especially macrophages, to infiltrate the vessel wall and further degrade vascular structural proteins. Further studies found that not only shrinkage of the aneurysm but also an increase in the thickness of the aneurysm wall was achieved by blocking NF-κB and MCP-1 activities at the first month after the formation of the experimental aneurysm. This may be achieved by reducing macrophage infiltration, decreasing MCP-1 expression, and restoring collagen biosynthesis [7]. Lai et al. found that in a rat aneurysm model, injection of the rats with APC-siRNA further activated the NF-κB signaling pathway and upregulated expression of MCP-1, TNF-α, IL-1, IL-6 MMP-2, and MMP-9, as well as enhanced the levels of p65 phosphorylation [8]. The above findings fully demonstrate that NF-κB pathway is involved in the development of intracranial aneurysms via regulating inflammatory response. Furthermore, miR-1246 may be also involved in the formation and rupture of intracranial aneurysms as a result of targeting and regulating the NF-κB pathway and triggering an inflammatory response.

Two important target genes, TP53 and GSK3B, were identified in this study. TP53 has been shown to be involved in the development of several cancers as a tumor suppressor, and TP53 was also found to have an important role in inflammation and inflammatory diseases. Li et al. analyzed two genotypes of TP53 and concluded that different genotypes of TP53 are capable of playing different roles in intracranial aneurysms. miR-34b/crs4938723 TT type has a higher incidence of intracranial aneurysms. TP53 is capable of mediating the inflammatory response involved in the formation of intracranial aneurysms [9]. GSK3B is not only one of the major regulators of the inflammatory response, but also a serine/threonine protein kinase signaling molecule that is widely expressed in many cell types [10]. The GSK3B/β-linked protein signaling pathway has been reported to promote the proliferation and migration of vascular smooth muscle cells. Higher phosphorylated GSK3B in atheroma leads to β-linked protein activation, and β-linked protein activation is involved in the resting transformation of vascular smooth muscle cells. This process has also been shown to be involved in arterial aging [11]. Analysis of transcription factors revealed that YY1 may be associated with the development of intracranial aneurysms. YY1 is a member of the Polycomb histone family, which acts as a typical multifunctional zinc finger transcription factor that can activate or repress biological processes such as gene transcription [12]. Zhou et al. found that YY1 plays a role in the endothelial inflammatory process by forming a functional complex through the NF-κB pathway and exerting transcriptional regulation on the inflammatory genes IL6 and IL8. The miRNAs, target genes, and their transcription factors in the above study are comparable with our predicted target genes, suggesting that these target genes may be the key genes in the regulatory pathways associated with the development and inflammation of the intracranial aneurysms [13].

## 5 Conclusion

In conclusion, a number of studies, including ours, suggest that inflammation is one of the most important factors in the development and rupture of aneurysm. Our study has shown that miR-1246 is highly expressed in patients with intracranial aneurysms and that our bioinformatic analysis indicates an important role of miR-1246 in regulating the inflammatory pathways, lipid and atherosclerotic signaling pathways. miR-1246 may serve as a potential diagnostic marker and therapeutic target for intracranial aneurysms.
